# Supplementary material for: End Sequence Analysis Toolkit (ESAT) expands the extractable information from single-cell RNA-seq data
Source: Genome Res. 2016 Oct;26(10):1397–410. doi: 10.1101/gr.207902.116 (PMC5052061; doi:10.1101/gr.207902.116)
Supplement: Supplemental Material [file supp_gr.207902.116_Supp_Materials.tar.gz › ESAT-esat_v0.1/doc/html/classbroad_1_1core_1_1siphy_1_1_conservation_utils.html]

ESAT: broad.core.siphy.ConservationUtils Class Reference


ESAT


- Main Page
- Related Pages
- Packages
- Classes
- Files

- Class List
- Class Index
- Class Hierarchy
- Class Members

All Classes Namespaces Files Functions Variables Enumerator Pages

- broad
- core
- siphy
- ConservationUtils

Static Public Member Functions |
List of all members

broad.core.siphy.ConservationUtils Class Reference

Collaboration diagram for broad.core.siphy.ConservationUtils:

[legend]

|  |  |
| --- | --- |
| Static Public Member Functions | |
| static void | setUninformativeNodes (Map< String, Matrix > column, List< String > sequences) |
|  | |
| static void | setUninformativeNodes (Map< String, Matrix > column, List< String > sequences, int colIdx) |
|  | |
| static Phylogeny | pruneTree (List< String > toPrune, Phylogeny tree) |
|  | |
| static Phylogeny | pruneTree (String toPrune, Phylogeny tree) |
|  | |
| static List< String > | getGappedSeqsInWindowMatrix (int window, Map< String, Matrix > alignmentMatrix, int start) |
|  | |
| static MultipleAlignment | setUpAlignment (ArgumentMap argMap, String alnFile, String alnFileFormat, List< String > ignoreList, EvolutionaryModel model) throws IOException, ParseException, FileNotFoundException |
|  | |
| static MultipleAlignment | setUpMAF (String alnFile, List< String > ignoreList, EvolutionaryModel model, int start, int end) throws IOException, ParseException, FileNotFoundException |
|  | |
| static MultipleAlignment | setUpMAF (MAFIO mafio, List< String > ignoreList, EvolutionaryModel model, int start, int end) throws IOException, ParseException, FileNotFoundException |
|  | |
| static MultipleAlignment | setUpMAF (MAFIO mafio, List< String > ignoreList, Phylogeny guideTree, int start, int end) throws IOException, ParseException, FileNotFoundException |
|  | |
| static List< String > | commaSeparatedStringToList (String csvListStr) |
|  | |

## Member Function Documentation

|  |  |  |  |  |  |
| --- | --- | --- | --- | --- | --- |
| static List<String> broad.core.siphy.ConservationUtils.commaSeparatedStringToList | ( | String | *csvListStr* | ) |  |

static

|  |  |  |  |
| --- | --- | --- | --- |
| static List<String> broad.core.siphy.ConservationUtils.getGappedSeqsInWindowMatrix | ( | int | *window*, |
|  |  | Map< String, Matrix > | *alignmentMatrix*, |
|  |  | int | *start* |
|  | ) |  |  |

static

|  |  |  |  |
| --- | --- | --- | --- |
| static Phylogeny broad.core.siphy.ConservationUtils.pruneTree | ( | List< String > | *toPrune*, |
|  |  | Phylogeny | *tree* |
|  | ) |  |  |

static

Here is the caller graph for this function:

|  |  |  |  |
| --- | --- | --- | --- |
| static Phylogeny broad.core.siphy.ConservationUtils.pruneTree | ( | String | *toPrune*, |
|  |  | Phylogeny | *tree* |
|  | ) |  |  |

static

Here is the call graph for this function:

|  |  |  |  |
| --- | --- | --- | --- |
| static void broad.core.siphy.ConservationUtils.setUninformativeNodes | ( | Map< String, Matrix > | *column*, |
|  |  | List< String > | *sequences* |
|  | ) |  |  |

static

|  |  |  |  |
| --- | --- | --- | --- |
| static void broad.core.siphy.ConservationUtils.setUninformativeNodes | ( | Map< String, Matrix > | *column*, |
|  |  | List< String > | *sequences*, |
|  |  | int | *colIdx* |
|  | ) |  |  |

static

|  |  |  |  |
| --- | --- | --- | --- |
| static MultipleAlignment broad.core.siphy.ConservationUtils.setUpAlignment | ( | ArgumentMap | *argMap*, |
|  |  | String | *alnFile*, |
|  |  | String | *alnFileFormat*, |
|  |  | List< String > | *ignoreList*, |
|  |  | EvolutionaryModel | *model* |
|  | ) |  | throws IOException, ParseException, FileNotFoundException |

static

Here is the call graph for this function:

|  |  |  |  |
| --- | --- | --- | --- |
| static MultipleAlignment broad.core.siphy.ConservationUtils.setUpMAF | ( | String | *alnFile*, |
|  |  | List< String > | *ignoreList*, |
|  |  | EvolutionaryModel | *model*, |
|  |  | int | *start*, |
|  |  | int | *end* |
|  | ) |  | throws IOException, ParseException, FileNotFoundException |

static

Here is the call graph for this function:

Here is the caller graph for this function:

|  |  |  |  |
| --- | --- | --- | --- |
| static MultipleAlignment broad.core.siphy.ConservationUtils.setUpMAF | ( | MAFIO | *mafio*, |
|  |  | List< String > | *ignoreList*, |
|  |  | EvolutionaryModel | *model*, |
|  |  | int | *start*, |
|  |  | int | *end* |
|  | ) |  | throws IOException, ParseException, FileNotFoundException |

static

Here is the call graph for this function:

|  |  |  |  |
| --- | --- | --- | --- |
| static MultipleAlignment broad.core.siphy.ConservationUtils.setUpMAF | ( | MAFIO | *mafio*, |
|  |  | List< String > | *ignoreList*, |
|  |  | Phylogeny | *guideTree*, |
|  |  | int | *start*, |
|  |  | int | *end* |
|  | ) |  | throws IOException, ParseException, FileNotFoundException |

static

Here is the call graph for this function:

---

The documentation for this class was generated from the following file:

- java/broad/core/siphy/ConservationUtils.java


---

Generated on Fri Aug 15 2014 15:10:56 for ESAT by  

 1.8.7
